# Dual roles of parathyroid hormone related protein in TGF-β1 signaling and fibronectin up-regulation in mesangial cells

**DOI:** 10.1042/BSR20171061

**Published:** 2017-10-27

**Authors:** Su-Zhen Wu, Si-Jun Yang, Hong-Min Chen, Fang-Fang Peng, Hong Yu, Joan C. Krepinsky, Bai-Fang Zhang

**Affiliations:** 1 Department of Biochemistry and Hubei Provincial Key Laboratory of Developmentally Originated Disease, Wuhan University School of Basic Medical Sciences, Wuhan, P.R. China; 2 Biochemistry Department, Gannan Medical University, Ganzhou, P.R. China; 3 ABSL-3 Laboratory at the Center for Animal Experiment and State Key Laboratory of Virology, Wuhan University School of Medicine, Wuhan, P.R. China; 4 Division of Nephrology, McMaster University, Hamilton, Ontario, Canada

**Keywords:** diabetic kidney disease, extracellular matrix, parathyroid hormone-related protein, TGF-β1

## Abstract

Little is known about the cross-talk between parathyroid hormone (PTH) related protein (PTHrP) and TGF-β1 in mesangial cells (MCs). Our results showed that PTHrP treatment (≤3 h) induced internalization of PTH1R (PTH/PTHrP receptor)–TβRII (TGF-β type 2 receptor) complex and suppressed TGF-β1-mediated Smad2/3 activation and fibronectin (FN) up-regulation. However, prolonged PTHrP treatment (12–48 h) failed to induce PTH1R–TβRII association and internalization. Total protein levels of PTH1R and TβRII were unaffected by PTHrP treatment. These results suggest that internalization of PTH1R and TβRII after short PTHrP treatment might not lead to their proteolytic destruction, allowing the receptors to be recycled back to the plasma membrane during prolonged PTHrP exposure. Receptor re-expression at the cell surface allows PTHrP to switch from its initial inhibitory effect to promote induction of FN. Our study thus demonstrates the dual roles of PTHrP on TGF-β1 signaling and FN up-regulation for the first time in glomerular MCs. These data also provided new insights to guide development of therapy for diabetic kidney disease (DKD).

## Introduction

Parathyroid hormone (PTH) related protein (PTHrP) is a widespread factor in fetal and adult tissues and plays a key role in the development and differentiation by paracrine/autocrine/intracrine pathways [1–3]. PTHrP and PTH show significant sequence homology, particularly within the first 13 amino acid residues [4]. PTH/PTHrP receptor (PTH1R) binds and is activated by the endocrine ligand PTH, as well as the paracrine ligand PTHrP [4–6]. PTH1R is highly expressed in bone and kidney and mediates the PTH-dependent regulation of mineral ion homeostasis [7–9]. PTHrP is also widely expressed in the kidney and exerts a modulatory action on renal function [10,11]. PTHrP is known to be up-regulated in several experimental nephropathies such as acute renal injury, diabetic kidney disease (DKD), and obstructive nephropathy [12–14], but the downstream pathophysiologic effects are still poorly understood.

TGF-β signaling is initiated by the formation of a heteromeric complex of specific receptors in the surface membrane of target cells. The TGF-β membrane receptor complex comprises two families of proteins with serine-threonine kinase activity, namely TGF-β type I receptor (TβRI) and TGF-β type 2 receptor (TβRII). TGF-β binds to TβRII, which then phosphorylates the Ser/Thr cluster of TβRI and activates TβRI, which in turn phosphorylates and activates several intracellular signaling cascades including Smads and MAPKs. These effectors regulate the transcription of TGF-β-responsive genes [15,16]. TGF-β has been recognized as an important profibrogenic mediator in chronic kidney diseases including DKD, which are characterized by the accumulation of extracellular matrix (ECM) and glomerulosclerosis [17–19].

Mesangial cell (MC) hypertrophy is one of the earliest morphologic changes in DKD [20]. Hypertrophic MCs secrete more ECM protein, resulting in expansion of the mesangial matrix [21]. A large body of evidence has demonstrated that TGF-β plays a key role in ECM accumulation [22–25]. Our previous study showed that short treatment of PTH inhibits the up-regulation of fibronectin (FN) and type IV collagen induced by TGF-β1 and high glucose (HG) in MCs [26]. Whether PTHrP is involved in ECM accumulation through TGF-β signaling has not been addressed. The present study investigated the interactions between PTHrP and TGF-β1 signaling in rat MCs and observed whether PTHrP has an effect on TGF-β1-induced ECM accumulation.

## Materials and methods

### Cell culture

Primary MCs were obtained from glomeruli of Sprague–Dawley rats (Center for Animal Experiment, Wuhan University, China) by differential sieving and grown in low-glucose DMEM (5.6 mM glucose) containing 20% FBS (Invitrogen, Carlsbad, U.S.A.). All cell culture media contained antibiotics (100 units/ml penicillin and 100 μg/ml streptomycin). Experiments were carried out using cells between passages 6 and 15. Confluent MCs were cultured in serum-free medium for 24 h prior to treatment. Glucose (24.4 mM) (final concentration: 30 mM) was added for HG levels. Recombinant human TGF-β1 (R&D Systems, Minneapolis, U.S.A.) was used at 2 ng/ml, PTHrP (1-34) peptide (Bachem, Bubendorf, Swiss) was used at 100 nM (here termed as PTHrP).

### Protein extraction and analysis

Protein was extracted from MCs with regular lysis buffer as published previously [27]. Briefly, cells were centrifuged for 10 min at 4°C, 10000×***g*** to pellet cell debris. After protein content was determined, 50 μg of total protein were separated on SDS/PAGE followed by Western blot. Antibodies included monoclonal PTH1R (1:1000, Sigma, St. Louis, U.S.A.), monoclonal TβRII (1:1000, Santa Cruz Biotechnology, Santa Cruz, U.S.A.), monoclonal FN (1:5000, BD Biosciences, San Jose, U.S.A.), polyclonal p-Smad2/3 (1:1000, Cell Signaling, Boston, U.S.A.), polyclonal Smad2/3 (1:1000, Cell Signaling), and monoclonal β-actin (1:5000, Sigma).

For cytosol and membrane fractionation, MCs were lysed with hypotonic lysis buffer as described previously [28]. Briefly, all lysates were homogenized using 25 G needle passage, and centrifuged at 4°C, 100000×***g*** for 1 h. Supernatant was collected as the cytosol fraction, and the precipitates were redissolved in regular lysis buffer containing 60 mM *N*-octyl-glucopyranoside. After centrifugation at 4°C, 100000×***g*** for 1 h, supernatant was collected as the membrane fraction.

For immunoprecipitation (IP) experiments, MCs were harvested in IP lysis buffer containing 60 mM *N*-octyl-glucopyranoside [29]. After cell lysis, equal amounts of protein lysate were incubated with 2 μg of PTH1R or TβRII primary antibody rotating overnight at 4°C, followed by 25 μl of protein G-agarose (Millipore, Massachusetts, U.S.A.) slurry for 90 min at 4°C. After rinsing with wash buffer, the immunoprecipitates were resuspended in 2× sample buffer, boiled for 5 min, and detected by Western blot.

### Transfection

Rat MCs were transfected with GFP-tagged TβRII at approximately 60% confluence as recently described [30]. The medium was changed to serum-free medium at 24 h, and MCs were then treated with 100 nM PTHrP after another 24 h. Fluorescence analysis was performed using a Leica TCS-SP2 confocal microscope.

### RNAi

Subconfluent MCs were transfected with 100 nM rat PTH1R siRNA or control siRNA (RiboBio, China) using Effectene Kit (Qiagen, Germany). The following two duplex sequences were used: sense 1, 5′-GGACGAUGUCUUUACCAAAdTdT-3′, antisense 1, 5′-UUUGGUAAAGACAUCGUCCdTdT-3′; and sense 2, 5′-GGCAGAUCCAGAUGCAUUAdTdT-3′, antisense 2, 5′-UAAUGAAUCUGGAUCCUGCCdTdT-3′. For experiments, 24 h after transfection, cells were made quiescent in 0% FBS for 24 h. MCs were then treated with TGF-β1 or HG for the indicated duration, with or without PTHrP. Cell lysates were harvested as described above. PTH1R protein level was analyzed by Western blot to assess the efficacy of down-regulation by RNAi.

### Statistical analysis

Statistical significance was tested with SPSS 20.0 through one-way ANOVA, with Tukey’s honestly significant difference (HSD) for post hoc analysis for experiments. Results are expressed as the means ± S.E.M. *P*<0.05 was considered significant. Experimental repetition times (*n*) are given in figure legends.

## Results

### Short PTHrP treatment attenuates TGF-β1/Smad signaling, while long PTHrP treatment induces FN up-regulation

**Figure 1:**
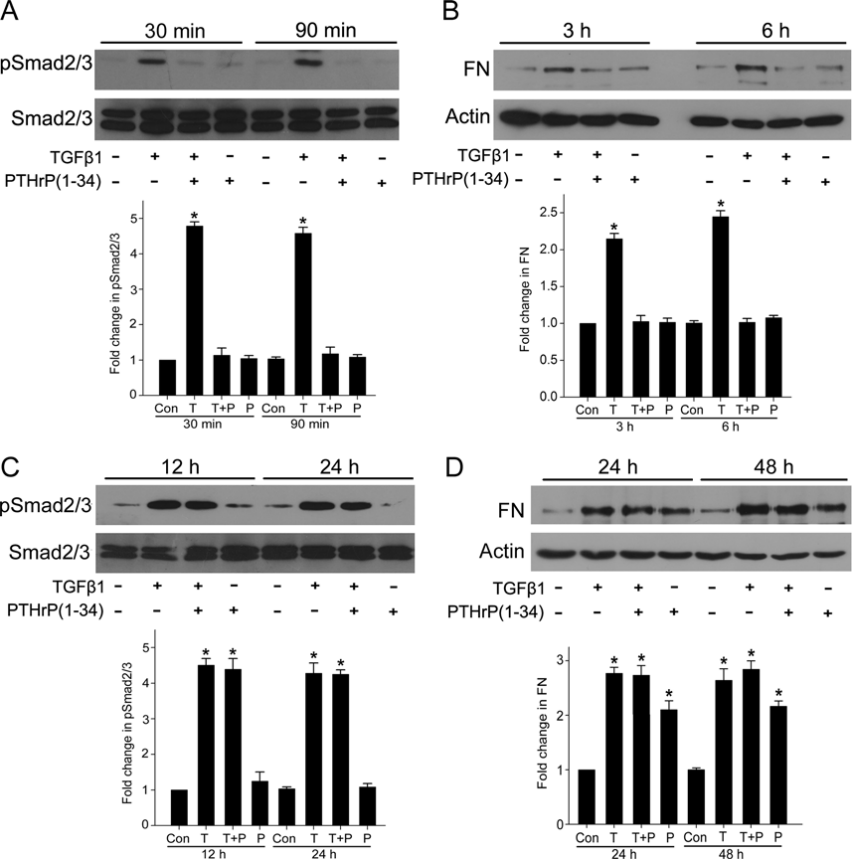
Short-term stimulation of PTHrP prevented TGF-β1/Smad signaling and FN up-regulation, whereas long-term treatment of PTHrP did not prevent TGF-β1-induced Smad2/3 phosphorylation and FN up-regulation MCs were pretreated with PTHrP (100 nM, 30 min) before TGF-β1 treatment for the indicated periods. (**A**,** C**) Smad2/3 phosphorylation was detected by Western blot (*P<0.05 compared with control, *n*=6). (**B**,** D**) The protein levels of FN were assessed by Western blot (*P<0.05 compared with control, *n*=5).

PTHrP and PTH show significant sequence homology. Since short PTH treatment inhibits TGF-β1 signaling in MCs [26], it suggests that PTHrP might attenuate the sensitivity of MCs to TGF-β1 in the short term. Indeed, PTHrP pretreatment decreased TGF-β1-induced phosphorylation of Smad2/3 within 90 min (Figure 1A). TGF-β1-induced up-regulation of FN within 6 h was also inhibited by PTHrP (Figure 1B). These results indicated that short PTHrP treatment attenuates TGF-β1/Smad signaling and ECM up-regulation in MCs. By contrast, prolonged PTHrP exposure (12–48 h) did not inhibit TGF-β1-induced Smad2/3 phosphorylation and FN up-regulation (Figure 1C,D). Interestingly, increased FN protein expression was seen after 24 h of PTHrP exposure alone (Figure 1D).

Numerous studies have demonstrated that HG-induced ECM up-regulation is mediated by TGF-β1 signaling [21], we therefore explored the role of PTHrP on HG-induced Smad2/3 activation and FN up-regulation in MCs. Short PTHrP treatment also inhibited HG-induced Smad2/3 phosphorylation and FN up-regulation, but prolonged PTHrP treatment had no inhibitory effect (Supplementary Figure S1). These results suggested that short and prolonged treatment of PTHrP might have different roles in MCs.

**Figure 2:**
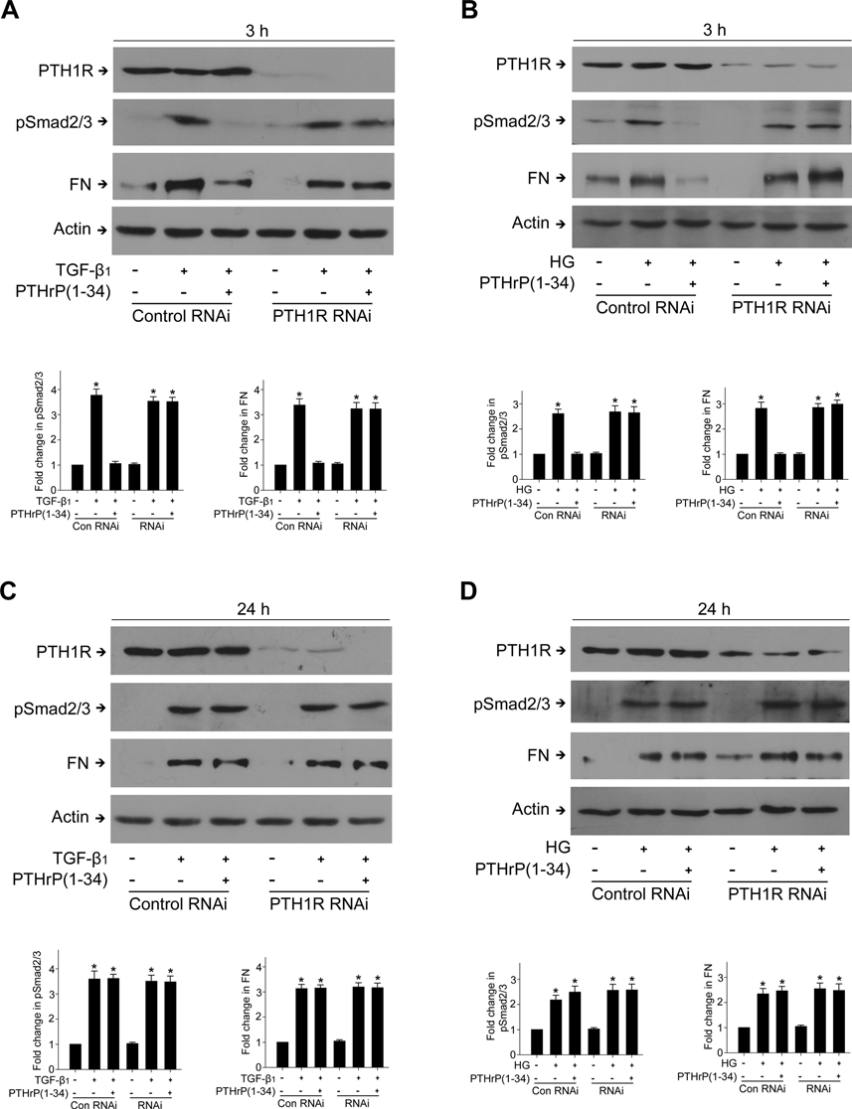
Knocking down PTH1R expression with siRNA reversed the blocking effect of PTHrP on TGF-β1 and HG-mediated Smad2/3 activation and FN up-regulation at 3 h, but not at 24 h Subconfluent MCs were transfected with 100 nM rat PTH1R siRNA or control siRNA. Cells were then treated with TGF-β1 or HG for the indicated duration, with or without PTHrP. The protein levels of p-Smad2/3 and FN were assessed by Western blot. (**A**)*P<0.05 compared with control, *n*=3. (**B**) *P<0.05 compared with control, *n*=3. (**C**) *P<0.05 compared with control, *n*=3. (**D**) *P<0.05 compared with control, *n*=3.

To elucidate the mechanism of the dual effects of PTHrP on TGF-β1 and HG-induced ECM up-regulation, we first examined the role of PTH1R using RNAi to down-regulate PTH1R. Figure 2 shows successful down-regulation of PTH1R protein by RNAi. Knocking down PTH1R reversed the blocking effect of PTHrP on early Smad2/3 activation and FN up-regulation (3 h) induced by TGF-β1 and HG (Figure 2A,B), indicating that PTH1R play an important role in the inhibition of TGF-β1 signaling mediated by PTHrP pretreatment. However, late Smad2/3 activation and FN up-regulation (24 h) induced by TGF-β1 and HG could not be reversed by PTH1R siRNA (Figure 2C,D).

### Although short PTHrP treatment induces endocytosis of PTH1R–TβRII complex in rat MCs, PTH1R and TβRII return to the plasma membrane during prolonged PTHrP treatment

**Figure 3:**
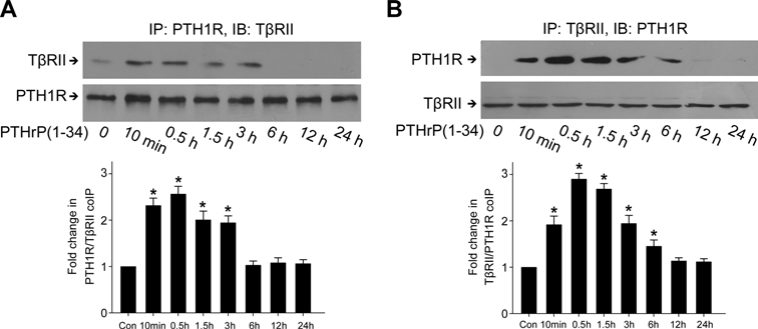
PTH1R formed complex with TβRII in the short-term stimulation of PTHrP, but not in the long-term treatment Serum-deprived rat MCs were treated with 100 nM PTHrP for the indicated duration. (**A**) IP of PTH1R was carried out and the protein levels of PTH1R and TβRII were assessed by Western blot. (**B**) IP of TβRII was carried out, then the protein levels of PTH1R and TβRII were assessed by Western blot (*P<0.05 compared with control, *n*=3).

**Figure 4:**
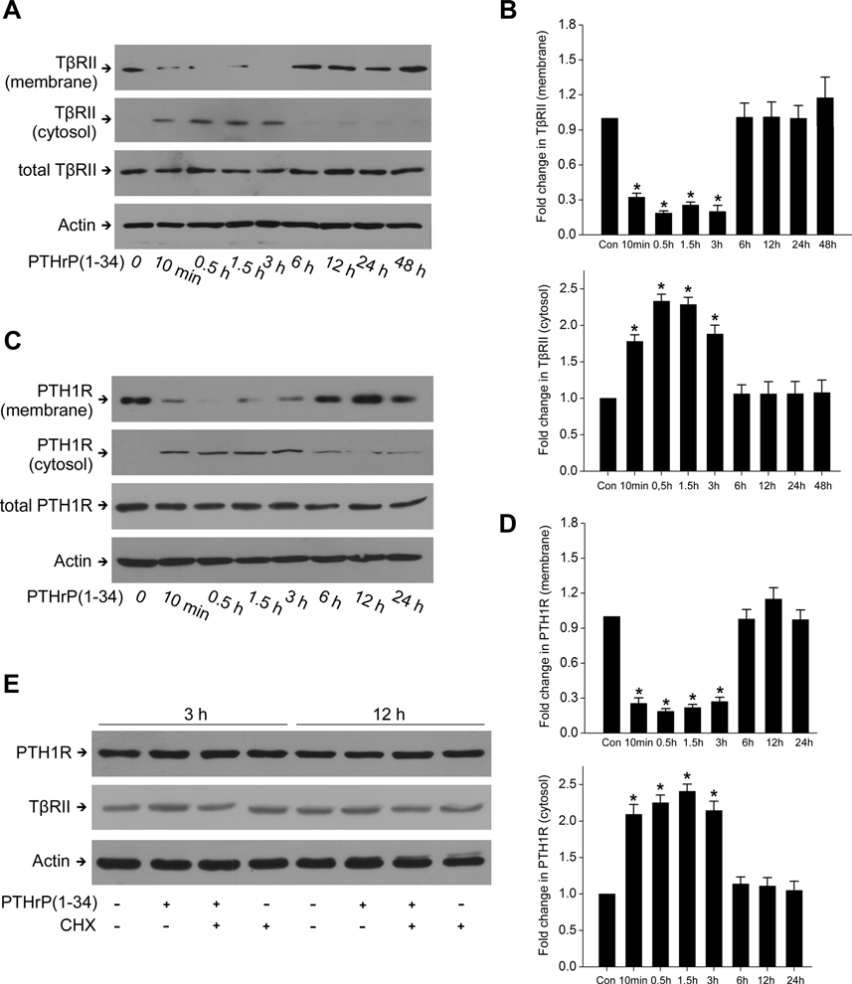
PTH1R and TβRII internalized into cytoplasm in the short-term stimulation of PTHrP, and returned to the cell surface after prolonged stimulus Serum-deprived rat MCs were treated with 100 nM PTHrP for the indicated time. (**A**,** B**) Western blot was performed to detect the protein level of TβRII in the cell membrane and cytoplasm, respectively (*P<0.05 compared with control, *n*=4). (**C**,** D**) Western blot was performed to detect PTH1R protein level in the cell membrane and cytoplasm, respectively (*P<0.05 compared with control, *n*=4). (**E**) MCs were pretreated with cycloheximide (100 ng/ml, 30 min) before PTHrP treatment for 3 or 12 h. The protein levels of PTH1R and TβRII were detected by Western blot with actin used as a loading control.

Given that PTH1R can be activated by PTH and PTHrP [4], we sought whether PTHrP alone induced the association between PTH1R and TβRII in rat MCs. As shown in Figure 3A,B, PTH1R specifically interacted with TβRII in response to PTHrP within 3 h in IP assays, suggesting that PTHrP induces the formation of a PTH1R–TβRII complex. The amount of cell surface PTH1R and TβRII decreased significantly from 10 min to 3 h, during which the amount of cytosolic PTH1R and TβRII increased (Figure 4A–D). These results showed that short PTHrP treatment induces the association of PTH1R with TβRII and internalization of this complex in rat MCs. However, prolonged PTHrP treatment (12–48 h) failed to induce PTH1R–TβRII association (Figure 3A,B) and internalization (Figure 4A–D). The protein levels of total PTH1R and TβRII did not reduce during PTHrP treatment (Figure 4A,B), indicating that internalized PTH1R and TβRII after short PTHrP treatment might not undergo proteolytic destruction.

**Figure 5:**
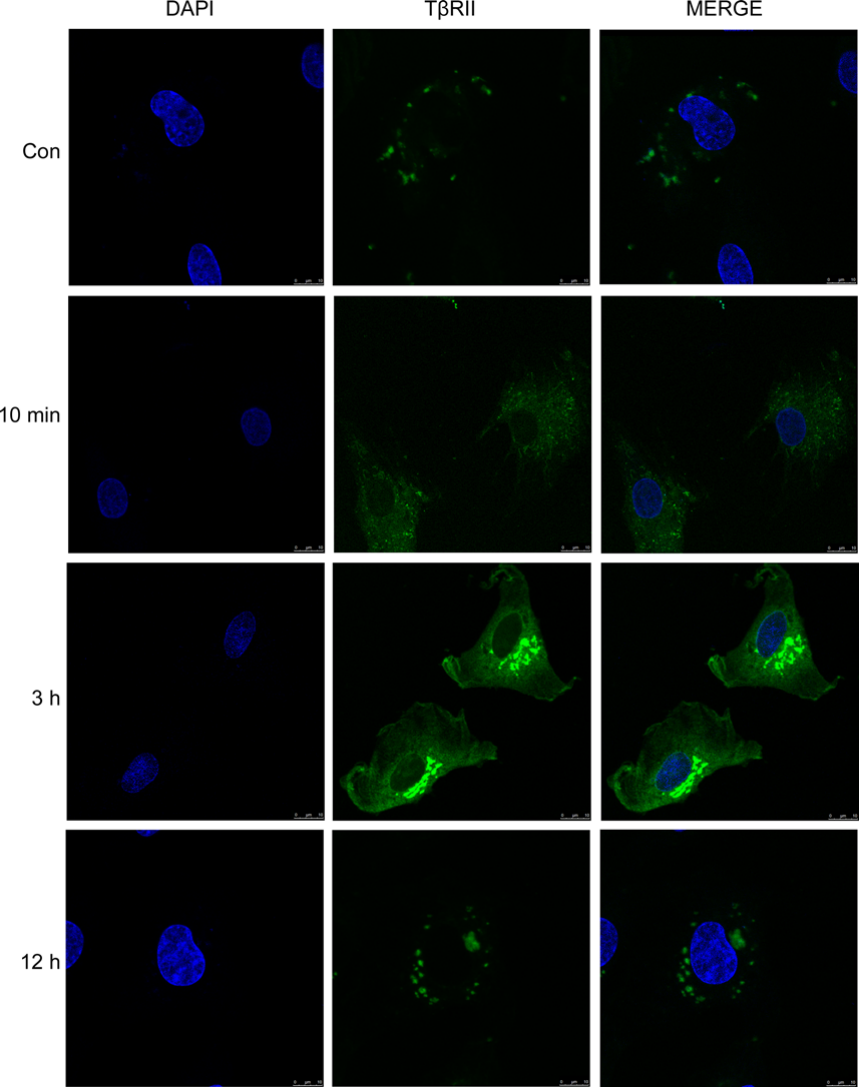
GFP-tagged TβRII was internalized into cytoplasm from 10 min to 3 h when stimulated with PTHrP alone, and returned to the plasma membrane at 12 h MCs were transfected with GFP-tagged TβRII for 24 h. Cells were then treated with 100 nM PTHrP for the indicated periods. Confocal microscopy was performed using Leica TCS-SP2. Scale bar, 10 µm.

To determine whether the internalized PTH1R and TβRII are recycled, we analyzed the effect of the protein synthesis inhibitor cycloheximide. Pretreatment with cycloheximide did not change the protein expression of total PTH1R and TβRII (Figure 4E), indicating that there is no *de novo* protein synthesis of PTH1R and TβRII. We next observed the location of TβRII by transfection with GFP-tagged TβRII. In the absence of PTHrP, TβRII was present predominantly at the cell surface. When stimulated with PTHrP, TβRII was internalized into cytoplasm from 10 min to 3 h. After prolonged PTHrP exposure, TβRII returned to the plasma membrane at 12 h (Figure 5). When put together, these results suggested that internalized PTH1R and TβRII may sort into a recycling pathway which mediates the return of these receptors to the cell surface.

## Discussion

Little is known about the cross-talk between PTHrP and TGF-β1 in the kidney. Previous studies showed that continuous expression of or incubation with PTHrP induced TGF-β up-regulation in MCs and podocytes [31,32]. The present study for the first time demonstrated the dual roles of PTHrP on TGF-β1 signaling. Our results showed that short PTHrP treatment induced the internalization of PTH1R–TβRII complex, decreased cell surface TβRII, and therefore inhibited TGF-β-mediated Smad2/3 activation and FN up-regulation in rat MCs, whereas prolonged PTHrP incubation could not suppress TGF-β/Smad signaling, but rather induced FN up-regulation.

It has been reported that prolonged exposure of cultured human MCs to PTHrP in the absence of exogenous growth factors induces a proliferative effect (24 h) followed by hypertrophy at 72 h [31,33,34], which is mediated by TGF-β1 [31]. The biological responses elicited by short PTHrP treatment are as yet unclear. We first examined whether short PTHrP treatment has an impact on TGF-β1/Smad pathway in rat MCs. Pretreatment with PTHrP prevented TGF-β1-induced Smad2/3 phosphorylation, and thereby inhibited the up-regulation of ECM protein FN within 6 h. Short PTHrP treatment also prevented HG-induced Smad2/3 phosphorylation and FN up-regulation. However, prolonged PTHrP exposure (12–48 h) failed to prevent HG and TGF-β1-induced Smad2/3 phosphorylation and FN up-regulation. Taking into account the importance of PTH1R as a critical initiator of PTHrP signals, we used PTH1R siRNA to confirm the role of PTH1R in the PTHrP and TGF-β1 signaling in MCs. Knocking down PTH1R reversed the blocking effect of PTHrP on early, but not late, Smad2/3 phosphorylation and FN up-regulation induced by TGF-β1 and HG. These data confirmed the important role of PTH1R in PTHrP-induced loss of TGF-β1 signaling at early time points.

We next investigated the interactions of PTH1R and TGF-β1 receptors. Our data showed that PTHrP alone induces the association of PTH1R with TβRII and the internalization of PTH1R and TβRII within 10 min. It is similar to those of previous studies showing that PTH induced the formation and internalization of PTH1R–TβRII complex within 30 min in osteoblasts [35] and MCs [26]. Short PTHrP treatment led to the loss of TGF-β1 signaling to Smad2/3 as a result of receptor internalization in rat MCs, with consequent inhibition of FN up-regulation. However, the fate of internalized receptors is still unknown. Internalized receptors may follow two classical intracellular pathways that determine later trafficking fate: (i) receptor sorting to the degradation pathway results in long-term attenuation of cellular responses; (ii) receptor sorting into a recycling pathway mediates return of functional receptors to the cell surface [36]. In the current study, cell membrane PTH1R and TβRII was recovered after 6 h, with a concurrent decrease in cytosolic PTH1R and TβRII. Moreover, there was no change in the total protein levels of PTH1R and TβRII after prolonged PTHrP exposure up to 24 h. In order to study the sorting machinery of PTH1R and TβRII, we used the protein synthesis inhibitor cycloheximide. Pretreatment with cycloheximide did not affect the expression of total PTH1R and TβRII. Put together, these results suggested that the PTHrP-mediated increase in cell membrane PTH1R and TβRII at 6 h did not require protein synthesis. PTH1R and TβRII may thus sort into a recycling pathway, mediating the return of both receptors to the plasma membrane. This is in accordance with results reported previously showing that PTH-induced internalized PTH1R does not undergo lysosomal degradation [37]. Since both PTH1R and TβRII returned to the cell membrane after prolonged PTHrP exposure, prolonged PTHrP exposure failed to prevent TGF-β1-induced Smad2/3 phosphorylation and FN up-regulation, indicating that the recycled TβRII receptors were functional. Although the underlying mechanism is not clear, prolonged PTHrP treatment (12–48 h) failed to induce PTH1R–TβRII association suggests that the separation of PTH1R–TβRII complex might involve in the functional recovery of both receptors. Further experiments are needed to elucidate the exact mechanisms underlying PTH1R and TβRII receptor recycling.

On the other hand, our results showed that PTHrP exposure alone significantly increased FN protein expression after 24 h, opposite to the early inhibiting effect on TGF-β1 or HG-induced FN up-regulation. This differential effect on FN protein expression could be attributed to the return of both PTH1R and TβRII to the plasma membrane at this later time point, allowing signaling through these receptors.

PTHrP peptide is recently used in the treatment of postmenopausal osteoporosis in human clinical trials and showing a probable security as a potential therapeutic agent [38,39]. Interestingly, short-term administration of PTHrP (1-36) results in bone formation. Whereas continuous infusion of PTHrP (1-36) results in marked and prolonged suppression of bone formation [40]. On the other hand, PTHrP is a very promising candidate for enhancing β-cell survival in diabetes mellitus [41–43]. Acute and systemic administration of PTHrP (1-36) peptide is capable of enhancing β-cell proliferation and mass in normal adult mice, without negatively affecting β-cell differentiation, size, and turnover [41]. In addition, short treatment of diabetic mice with PTHrP (1-36) restores bone mass and strength [44]. These findings support the therapeutic potential of acute treatment of amino-terminal PTHrP peptide for diabetes mellitus and diabetes-related complication such as DKD. Combined with the paradoxical roles of acute and chronic treatment of PTHrP in bone, our findings indicated that acute and chronic systemic administration of PTHrP has different effects on the diabetic kidney. Short-term administration of PTHrP peptide might have renoprotective effects due to decreased activities of TGF-β signaling in DKD, whereas chronic administration of PTHrP promotes ECM accumulation mediated by TGF-β1 in diabetic rats.

In conclusion, short PTHrP treatment induces the internalization of the PTH1R–TβRII complex and prevents HG and TGF-β-mediated Smad2/3 activation and FN up-regulation in rat MCs. However, internalized receptors return to the cell surface after prolonged PTHrP exposure and mediate PTHrP-induced FN up-regulation. The present study provides the first evidence of dual roles for PTHrP in TGF-β signaling and ECM up-regulation in MCs. Further studies need to be performed in order to illuminate the interactions between PTHrP and the TGFβ1 pathway *in vivo*. It might be helpful to guide the development of therapy for DKD.

## Supporting information

## Abbreviations

DKD: diabetic kidney disease
ECM: extracellular matrix
FN: fibronectin
HG: high glucose
IP: immunoprecipitation
MC: mesangial cell
PTH: parathyroid hormone
PTHrP: PTH related protein
PTH1R: PTH/PTHrP receptor
TβRI: TGF-β type I receptor
TβRII: TGF-β type 2 receptor
