# Supplementary figures and images for: Dual roles of parathyroid hormone related protein in TGF-β1 signaling and fibronectin up-regulation in mesangial cells
Source: Biosci Rep. 2017 Oct 27;37(5):BSR20171061. doi: 10.1042/BSR20171061 (PMC5665616; doi:10.1042/BSR20171061)

A

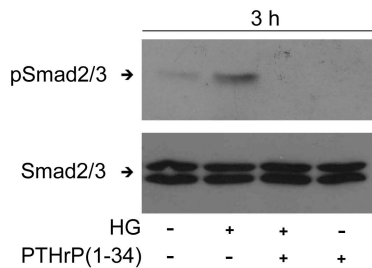

B

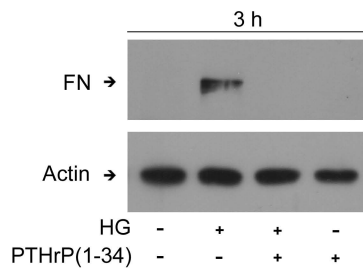

C

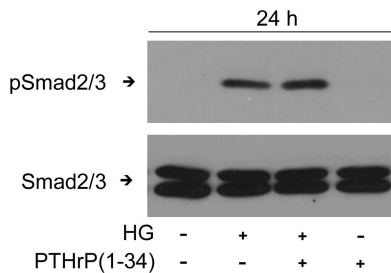

D

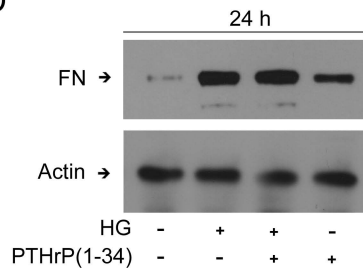

Supplement: Supplementary file 1 [file BSR20171061_Supp1.pdf]
